# Hippo-YAP1 Is a Prognosis Marker and Potentially Targetable Pathway in Advanced Gallbladder Cancer

**DOI:** 10.3390/cancers12040778

**Published:** 2020-03-25

**Authors:** Patricia García, Lorena Rosa, Sergio Vargas, Helga Weber, Jaime A. Espinoza, Felipe Suárez, Isabel Romero-Calvo, Nicole Elgueta, Vanessa Rivera, Bruno Nervi, Javiera Obreque, Pamela Leal, Eduardo Viñuela, Gloria Aguayo, Sabrina Muñiz, Alfredo Sagredo, Juan C. Roa, Carolina Bizama

**Affiliations:** 1 Department of Pathology, School of Medicine, Pontificia Universidad Católica de Chile, Santiago 8330024, Chile; pgarciam@uc.cl (P.G.); l.rosa01@ufromail.cl (L.R.); felipe.suarez@uc.cl (F.S.); nelguetav@hospitaldetalca.cl (N.E.); uzume278@gmail.com (V.R.); jaobreque@uc.cl (J.O.); sagredo1986@ug.uchile.cl (A.S.); 2 Applied Molecular and Cellular Biology PhD Program, Universidad de La Frontera, Temuco 4811230, Chile; 3 Department of Hematology Oncology, School of Medicine, Pontificia Universidad Católica de Chile, Santiago 8331150, Chile; ssvargas@uc.cl (S.V.); bnervi@med.puc.cl (B.N.); mamuniz@uc.cl (S.M.); 4 Centre of Excellence in Translational Medicine (CEMT) and Scientific and Technological Bioresource Nucleus (BIOREN), Universidad de la Frontera, Temuco 4810296, Chile; helga.weber@ufrontera.cl (H.W.); pamela.leal@ufrontera.cl (P.L.); 5 SciLifeLab, Division of Genome Biology, Department of Medical Biochemistry and Biophysics, Karolinska Institutet, Solna, Stockholm 17165, Sweden; jaime.espinoza.ruiz@ki.se; 6 Biomedical Visualization Graduate Program, Department of Biomedical and Health Information Sciences. College of Applied Health Sciences. University of Illinois at Chicago, Chicago, IL 60607, USA; iromer5@uic.edu; 7 Department of Digestive Surgery, Hepato-Bilio-Pancreatic Surgery Unit, Surgery Service, Complejo Asistencial Hospital Dr. Sótero del Río, Santiago 8207257, Chile; evinuela@uc.cl; 8 Department of Pathology, Complejo Asistencial Hospital Dr. Sótero del Río, Santiago 8207257, Chile; gaguayo@ssmso.cl; 9 Millennium Institute on Immunology and Immunotherapy, Pontificia Universidad Católica de Chile, Santiago 8331150, Chile

**Keywords:** gallbladder cancer, hippo pathway, molecular-targeted therapies, YAP1, verteporfin, patient-derived organoids

## Abstract

Gallbladder cancer is an aggressive disease with late diagnosis and no efficacious treatment. The Hippo-Yes-associated protein 1 (YAP1) signaling pathway has emerged as a target for the development of new therapeutic interventions in cancers. However, the role of the Hippo-targeted therapy has not been addressed in advanced gallbladder cancer (GBC). This study aimed to evaluate the expression of the major Hippo pathway components mammalian Ste20-like protein kinase 1 (MST1), YAP1 and transcriptional coactivator with PDZ-binding motif (TAZ) and examined the effects of Verteporfin (VP), a small molecular inhibitor of YAP1-TEA domain transcription factor (TEAD) protein interaction, in metastatic GBC cell lines and patient-derived organoids (PDOs). Immunohistochemical analysis revealed that advanced GBC patients had high nuclear expression of YAP1. High nuclear expression of YAP1 was associated with poor survival in GBC patients with subserosal invasion (pT2). Additionally, advanced GBC cases showed reduced expression of MST1 compared to chronic cholecystitis. Both VP treatment and YAP1 siRNA inhibited the migration ability in GBC cell lines. Interestingly, gemcitabine resistant PDOs with high nuclear expression of YAP1 were sensitive to VP treatment. Taken together, our results suggest that key components of the Hippo-YAP1 signaling pathway are dysregulated in advanced gallbladder cancer and reveal that the inhibition YAP1 may be a candidate for targeted therapy.

## 1. Introduction

The gallbladder cancer (GBC) is the seventh most common gastrointestinal cancer worldwide, with an incidence of 2.3/100,000 and mortality rate of 1.7/100,000 [1]. Advanced or metastatic GBC is associated with late diagnosis, unsatisfactory treatment and poor prognosis.

Given the few therapeutic options for advanced GBC, new biomarkers and therapeutic approaches must be explored in order to direct rational therapies to improve outcomes. The Hippo-Yes-associated protein 1 (YAP1) signaling pathway is dysregulated in many different cancers and has recently emerged as a master regulator that playing a central role in tumorigenesis, regulation of apoptosis, acquisition of tumor stem cell phenotype, drug resistance and metastatic potential [2]. The key components of this pathway include an upstream kinase cascade of serine/threonine mammalian Ste20-like protein kinases 1 and 2 (MST1/MST2) and large tumor suppressor 1 and 2 (LATS1 y LATS2) and the major effectors, the transcriptional regulators YAP1 and TAZ (transcriptional coactivator with a PDZ-binding domain) [3,4]. When the upstream kinases are inactivated, dephosphorylated YAP1/TAZ translocated into the nucleus and induce the expression of target genes such as *BIRC5* (Survivin), *CTGF*, *AREG*, *AXL* [3,4,5,6,7]. However, little is known about the status of the Hippo pathway in GBC. Li et al., reported for the first time that nuclear YAP1 is expressed in most GBC cases and that high nuclear YAP1 expression was associated with advanced tumor stage and worse patient survival. Consistently, knockdown of *YAP1* by shRNA in GBC-SD and OCUG-1 gallbladder cells significantly inhibited cell proliferation and invasion in vitro and inhibited cancer cell growth in vivo [6].

Verteporfin (VP) also known as Visudyne is a FDA approved drug used as a photosensitizer for photodynamic therapy in patients with age-related macular degeneration, but also inhibits YAP1 transcriptional activity, independent of light activation [8,9,10]. Currently, VP is the main pharmacological tool to understand the role of YAP1 and a therapeutic alternative for various cancers with a dysregulated Hippo pathway and high nuclear expression of YAP1 [8,9,10,11,12,13,14]. However, no reports have evaluated the potential therapeutic role of this YAP1 inhibitor in advanced GBC.

Here, we determined the expression levels of the main components of the Hippo-YAP1 signaling pathway YAP1, TAZ and MST1 in GBC and chronic cholecystitis patients. Further, we evaluated changes in migration/invasion capacities after YAP1 inhibition by siRNA and VP treatment in metastatic GBC cell lines. Finally, we evaluated the potential cytotoxicity effect of VP in patient-derived organoids (PDOs) as a preclinical model of advanced GBC.

## 2. Results

### 2.1. Key Components of the Hippo-YAP1 Pathway Are Dysregulated in Advanced Gallbladder Cancers

To evaluate the Hippo-YAP1 signaling pathway status in GBC, we performed an exploratory analysis using eight advanced GBC and eight chronic cholecystitis (CC) patient samples. The expression levels of Hippo pathway core components including *STK3/STK4* (MST1/2), *LATS1/2, SAV1, MOB1A/1B, YAP1* and *TAZ* were characterized by RT-qPCR. From these results, we found that transcript expression of *SAV1* was downregulated by 1.65-fold (*p* = 0.003), *LATS2* by 1.95-fold (*p* = 0.010) and *YAP1* by 1.4-fold (*p* = 0.007) in tumor samples compared with CC samples (Figure S1). Furthermore, mRNA profiling in GBC cell lines was consistent with these observations. Based on these findings and following antibody validation for immunohistochemistry (IHC) in paraffin-embedded samples, we performed IHC for the transcriptional co-activators YAP1/TAZ and the tumor suppressor gene MST1, an upstream kinase which activates LATS2, in an extended cohort of GBC and CC samples (as a non-cancerous control). We also analyzed the expression of these markers in dysplasia and incipient gallbladder cancer samples (Table S1).

**Figure 1:**
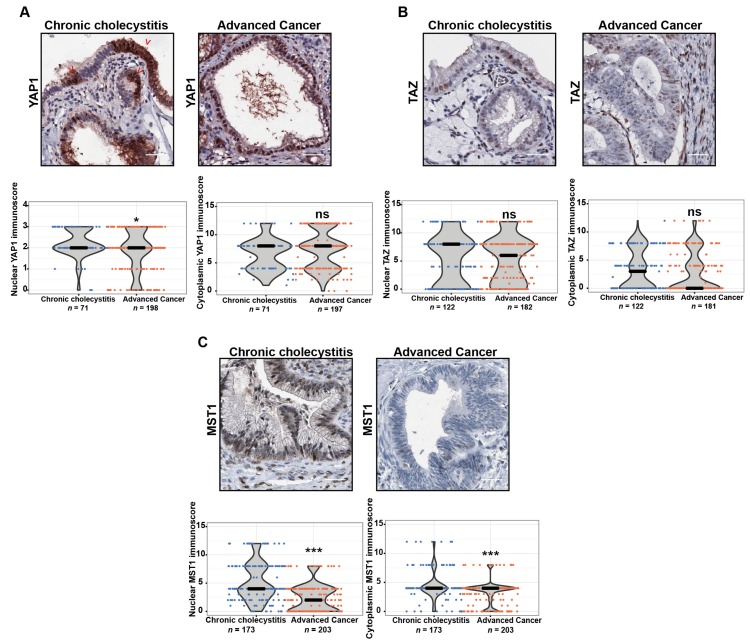
Expression of Hippo-Yes-associated protein 1 (YAP1) signaling pathway-related proteins in gallbladder cancer (GBC) patients. Representative immunohistochemistry images and results of staining scores (immunoscore) in chronic cholecystitis and advanced cancer cases (cytoplasmic and nuclear staining scores): (**A**) YAP1; (**B**) TAZ; (**C**) MST1 protein expression in chronic cholecystitis and in a well differentiated pT2 advanced cancer. Red arrows indicated the patchy staining pattern in YAP1. Scale bar in immunohistochemistry assays: 40 µm. The immunoscores are represented as median with rank (*** *p* < 0.001 by Wilcoxon–Mann–Whitney U-Test).

Given that nuclear localization of YAP1 and TAZ is critical for the oncogenic role of both proteins, we assessed nuclear and cytoplasmic expression separately. The analysis showed high nuclear immunoreactivity (positive staining score ≥ 2) of both proteins in CC and GBC tissues. Specifically, high nuclear and cytoplasmic YAP1 immunoscores were detected in 70.7% (169/239) and 63.1% (125/216) of advanced cancers and 91.5% (65/71) and 100% (71/71) of CC samples, respectively (Figure 1A, Table S1). The observed immunoscores for nuclear YAP1 showed that global protein expression of YAP1 was reduced in advanced GBC compared with CC (Figure 1A, *p* < 0.0363). Similarly, RT-qPCR results for YAP1 were consistent with our observations in IHC staining (Figure S1). However, two distinctive patterns of both nuclear and cytoplasmatic YAP1 expression were observed in 36.5% (27/74) of the CC samples, a patchy staining (Figure 1A) or a strong expression only in the deeper portions of the epithelia. Interestingly, this pattern was also observed in four chronic cholecystitis patient derived organoids (CC-PDOs) (Figure S2), where a high cytoplasmic YAP1 expression was observed in 80%–100% of epithelial cells from PDOs. Meanwhile, the expression of nuclear YAP1 was more variable, ranging from 40%–80% of the epithelial cells. The expression of YAP1 did not correlate with any of the clinicopathological features evaluated (Table S1). On the other hand, high nuclear and cytoplasmic TAZ immunostaining were found in 40.1% (73/132) and 65.4% (119/182) of advanced cancers and 67.2% (82/122) and 49.2% (50/122) of CC samples, respectively (Figure 1B, Table S1). Considering the observed immunoscores for both cytoplasmic and nuclear TAZ scores, we found no differences in the global protein expression of TAZ in advanced GBC compared with CC (Figure 1B). High expression of TAZ was significantly associated with higher pT stage, both the nuclear (*p <* 0.001) and cytoplasmic (*p <* 0.000) (Table S1).

Considering the observed immunoscores for both cytoplasmic and nuclear MST1 scores, global protein expression of MST1 was significantly reduced in advanced GBC compared with CC (Figure 1C, *p* < 0.001). We did not observe an association between the cytoplasmic expression of MST1 and the clinicopathological characteristics of the patients. Nevertheless, low nuclear MST1 expression (< 4 staining score) was significantly correlated with a higher pT stage (*p* = 0.004) (Table S1). Moreover, nonparametric analysis using Jonckheere-Terpstra test showed a significant decreasing trend in nuclear MST1 IHC score across CC, dysplasia, incipient and advanced GBC samples (*p* = 0.0012).

### 2.2. Nuclear Expresion of YAP1 Correlated with Poor Prognosis in Subserosal Gallbladder Cancers (pT2)

Two hundred and thirty-six samples were accrued from patients with confirmed GBC diagnosis and clinicopathological records, with a median follow-up of 11 months (range 1.0–271.9 months).

**Figure 2:**
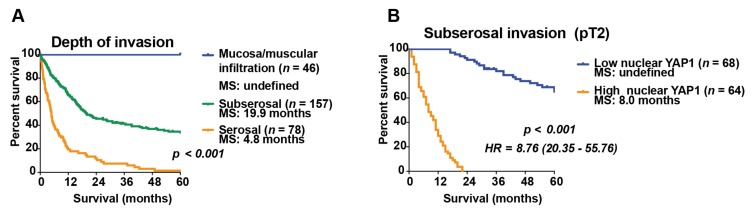
Nuclear expression of YAP1 in GBC is the second most important prognostic factor after depth of invasion in patients with subserosal infiltration (pT2). (**A**) GBC patient survival rates by tumor infiltration. Subserosal infiltration had an intermediate prognostic value (median survival 19.9 months) by univariate analysis; (**B**) Subserosal gallbladder cancer patient survival rates by high and low YAP1 nuclear expression. Patients with subserosal GBC and high nuclear YAP1 had a median survival of 8.0 months compared to patients with subserosal GBC and low nuclear YAP1, which have undefined survival by multiple correlation analyses. MS: Median survival; HR: Hazard ratio. 95% Confidence interval (0.80–1.86 months).

Clinicopathological variables, including IHC markers, were analyzed by multiple correlations test in a logarithmic multinomial model in order to assess independency between the variables. In accordance with a previous report by Cai et al., [15] we found that only tumor infiltration was significantly associated with median survival (Table S2). Next, we performed univariate analysis to stratify patients according to tumor infiltration grade. Patients with mucosa/muscular infiltration showed excellent overall survival rates (no deaths within the first 5 years of follow-up) compared to those with serosal infiltration (median survival 4.8 months, *p* < 0.001). Interestingly, patients with subserosal infiltration (pT2) had an intermediate prognosis (median survival 19.9 months), different to both mucosa/muscular and serosal infiltration (Figure 2A).

Therefore, additional prognostic markers were specifically analyzed in this subgroup of patients. A new multiple correlation analysis was performed considering immunohistochemical markers, showing that high YAP1 nuclear expression predicts poor survival among GBC patients with subserosal infiltration (Table S3). Next, a survival probability function was calculated by using the Cox hazard model, including only YAP1 nuclear expression within the survival equation. Interestingly, the median survival of patients with subserosal infiltration and high YAP1 nuclear expression (represented by a Cox survival probability greater than or equal to 0.5) was comparable with serosal-infiltrating tumors (median survival 8.0 months). Conversely, median survival of patients with subserosal infiltration and low YAP1 nuclear expression (represented by a Cox survival probability lower than 0.5) was comparable with mucosa/muscular-infiltrating tumors (undefined median survival = survival exceeded 50% at the longest time point and the median survival could not be computed) (Figure 2B; the variables used in the Cox proportional hazard regression analysis are available in Table S4).

### 2.3. YAP1 Knockdown Inhibits Migration of GBC Cell Lines

**Figure 3:**
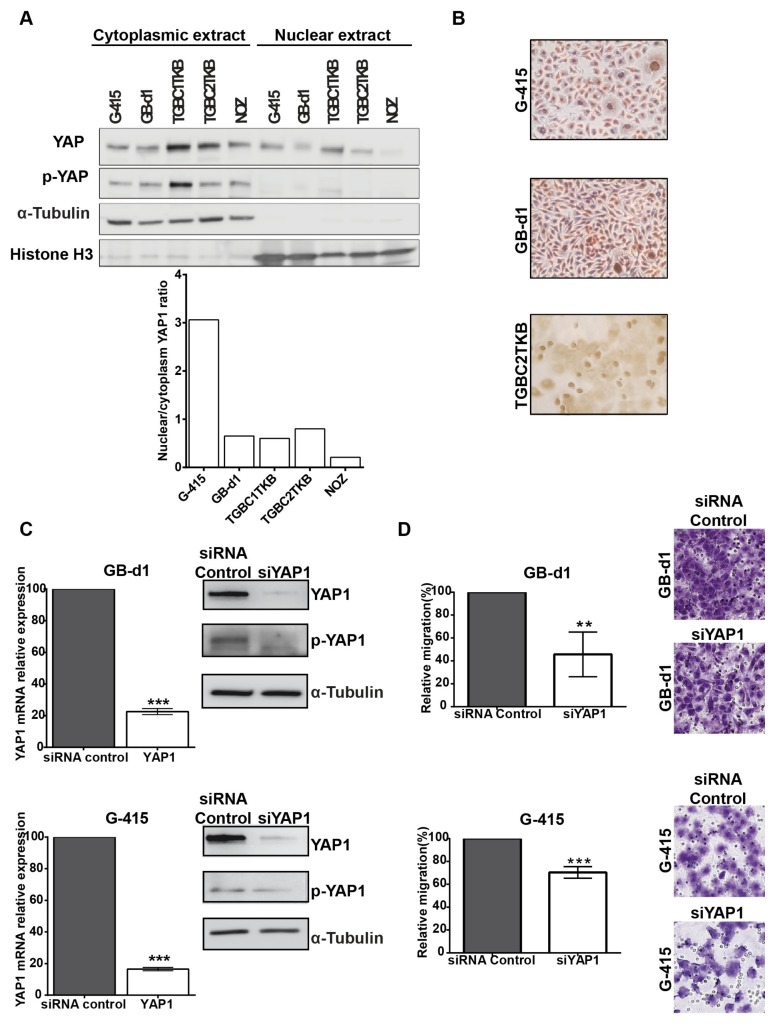
Knockdown of YAP1 by siRNA affects the migration capacities in GB-d1 and G-415 gallbladder cancer cell lines. (**A**) Cytoplasmic and nuclear protein expression of YAP1 and phospho-YAP1 in GBC cell lines by Western blot and estimation of the YAP1 nuclear/cytoplasmic ratio. Normalized expression of cytoplasmic phospho-YAP1 and nuclear YAP1 was quantified by densitometry. (**B**) Immunocytochemistry of YAP1 in GBC cell lines (Magnification: 20×). GB-d1 and G-415 cancer cells were transfected with 25nM of siRNA against YAP1 or siRNA non-target control; (**C**) the knockdown of YAP1 was validated by assessing mRNA and protein levels in GB-d1 G-415 cell lines at 24 h. YAP1 was quantified by qRT-PCR using *QARS* and *TFCP2* as internal controls, while α-tubulin was used as an internal control for loading protein. (**D**) Migration analysis of GB-d1 and G-415 gallbladder cancer cells treated for 24 h with siRNA non-target control and siRNA YAP1, *n* = 3. ** *p* < 0.01 and *** *p* < 0.001 by one-sample *t-*test. Magnification: 20×.

To perform functional analyses of YAP1, we first evaluated YAP1 protein expression at cytoplasmic and nuclear level in 5 human GBC cell lines: G-415, GB-d1, TGBC1TKB, TGBC2TKB and NOZ. We calculated a nuclear/cytoplasm ratio, which showed that G-415, TGBC2TKB and GB-d1 exhibited the highest YAP1 nuclear expression (Figure 3A). We decided to use G-415 and GB-d1 because YAP1 nuclear expression was present in 100% of both cells in comparison with TGC2TKB, of which only 50% of the cells showed nuclear expression (Figure 3B).

In order to silence *YAP1* gene in GB-d1 and G-415 GBC cell lines, we optimized a transfection protocol using a pool of 4 siRNAs targeting *YAP1* at a concentration of 25 nM for 24 h (Figure S3). We next evaluated the efficiency of the *YAP1* knockdown in GB-d1 and G-415 cells. Targeting *YAP1* expression with siRNA pool reduced *YAP1* mRNA in GB-d1 and G-415 cell lines by 77.4% (*p* = 0.0002) and 83.5% (*p* < 0.0001), respectively. This was accompanied by a marked reduction of YAP1 and phospho-YAP1 protein expression by western blot (Figure 3C). Moreover, we found that decreased expression of YAP1, by siRNA pool transfection, significantly reduced the migration capacity of GB-d1 and G-415 cancer cells to 54.3% (*p* = 0.0404) and 30.0% (*p* = 0.0097) compared to control siRNA-transfected cells, respectively (Figure 3D). The decreased expression of YAP1 does not significantly affect the invasion capacity in GB-d1 and G-415 cells (Figure S4).

### 2.4. The YAP1 Inhibitor, Verteporfin, Reduced Migration and Invasion in GBC Cells Lines

**Figure 4:**
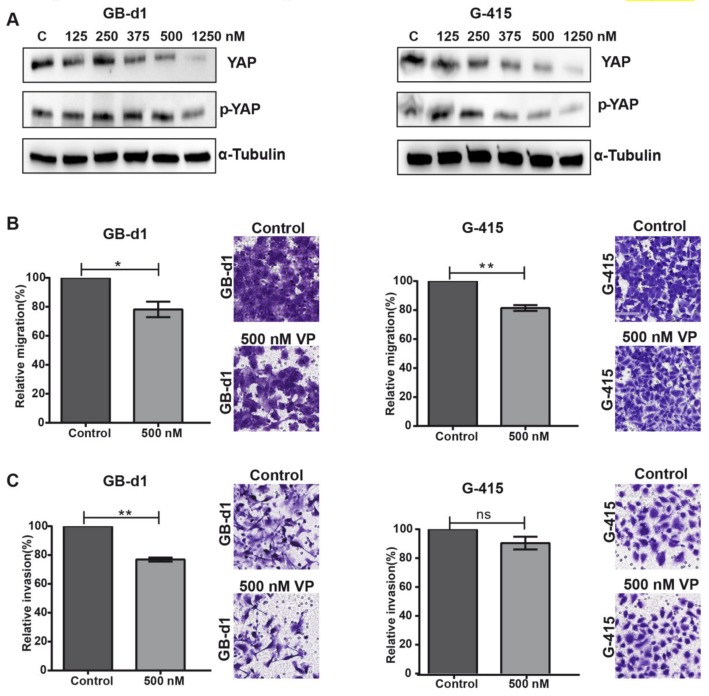
In vitro treatment with Verteporfin reduces migration and invasion capabilities in GB-d1 and G-415 cell lines. (**A**) Reduced expression of YAP1 and phospho-YAP in GB-d1 and G-415 cells treated with increasing concentrations of Verteporfin (VP). (**B**) Relative migration of GB-d1 and G-415. Magnification: 20×. (**C**) Relative invasion of GB-d1 and G-415. Results are represented as Mean ± SD from 3 independent experiments (* *p* < 0.05, ** *p* < 0.01 and ns: no significant by one-sample *t*-test). Magnification: 20×.

After initial evaluation of the biological effects of YAP1 inhibition via siRNA pool on GBC cell lines, we studied the in vitro effects of VP, a known suppressor of YAP1-TEAD complex with great potential to be used in cancer therapy [16]. We examined protein expression of YAP1 and phospho-YAP in GB-d1 and G-415 cell lines treated with increasing concentrations of VP (125–1250 nM) at 48 h by western blot assay. VP reduced YAP1 and phospho-YAP1 protein levels in both GBC cell lines in a dose-dependent manner, compared to DMSO-treated control (Figure 4A).

To further explore the potential effects of VP on the migratory and invasive capacities of GBC cancer cells, GB-d1 and G-415 cells were exposed to 0.004% DMSO (vehicle) and 500 nM or 1250 nM of VP for 24 h (migration assay) and 48 h (invasion assay). As shown in Figure 4B, migration was significantly reduced by 21.8% (*p* = 0.0195) in GB-d1 cells and by 18.6% (*p* = 0.0038) in G-415 cells compared with the vehicle control. Moreover, a significant reduction of invasiveness was found only in GB-d1 cells treated with VP (23.1% reduction, *p* = 0.0012, Figure 4C).

Next, we evaluated cell viability by MTS using increasing VP concentrations, from 125 to 500 nM. Treatment with VP did not affect cell viability (Figure S5). Additionally, we investigated the effects of VP on apoptosis and cell cycle dynamics by flow cytometry analysis. These results indicated that exposure of 500 nM VP did not affect apoptosis or the cell cycle in either GB-d1 or G-415 cells at 24 h or 48 h (Figure S6). Taken together, our results indicated that VP can effectively hinder migration and invasion in GBC cells.

### 2.5. Gemcitabine-Resistant GBC Patient-Derived Organoids Are Sensitive to Verteporfin Treatment

**Figure 5:**
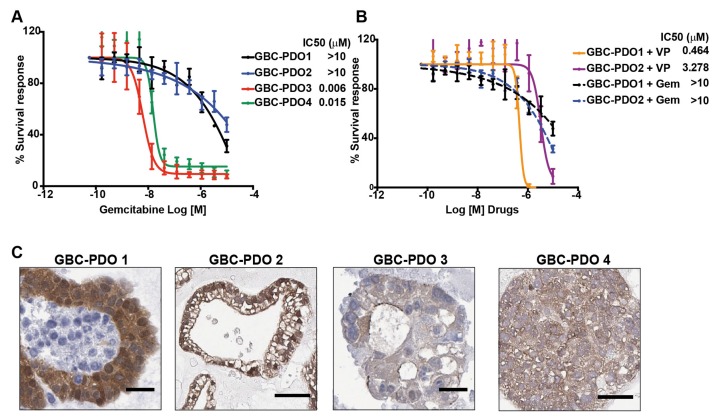
Gemcitabine-resistant gallbladder cancer patient-derived organoids (GBC-PDOs) are sensitive to Verteporfin treatment. (**A**) Dose-response curves to gemcitabine generated in GBC-PDOs. GBC-PDOs 1 and 2 are resistant to gemcitabine while GBC-PDOs 3 and 4 are sensitive to gemcitabine, *n* = 3. (**B**) Dose-response curves to VP generated in gemcitabine-resistant GBC-PDOs (GBC-PDO1 and GBC-PDO2), *n* = 3. These plots showed the percentage of viable cells measured by CellTiter Glo assay in response to micromolar doses (data expressed as Log10) at 72 h of treatment with each drug. Results are represented as Mean ± SEM. (**C**) Representative image of YAP1 expression in GBC-PDOs 1, 2, 3 and 4. Scale bar: 50 µm.

We further studied the inhibitory properties of VP in a pre-clinical model of GBC patient-derived organoids (PDOs). Organoids are 3D structures propagated from epithelial stem cells that exist within the tumor tissue, by culture them in a medium that favors undifferentiated cell enrichment [17,18]. They have an indefinite passaging capability, genetic stability and recapitulate the heterogeneity of tumor tissues and patient responses in the clinic, thereby representing a valuable and feasible pre-clinical model for using in personalized medicine [19]. Here, we established PDOs from gallbladder tumors and treated them with gemcitabine, a chemotherapeutic agent used in GBC, for testing dosage-dependent response. The drug response curves showed that GBC-PDO1 and GBC-PDO2 exhibited high resistance to gemcitabine treatment compared to GBC-PDO3 and GBC-PDO-4, as indicated by the half maximal inhibitory concentration (IC_50_) (Figure 5A). Thus, the effect on cell viability of VP was evaluated in gemcitabine-resistant PDOs (GBC-PDO1 and GBC-PDO2) as an alternative therapeutic strategy. Both PDOs showed high sensitivity against VP compared to gemcitabine, with no viable cells after 72 h of drug treatment (Figure 5B). Interestingly, GBC-PDO1 and GBC-PDO2 had higher nuclear expression of YAP1 (positive staining 2 and 3, respectively) than GBC-PDO3 and GBC-PDO4 (Figure 5C).

Next, we evaluated if additive cytotoxic effects occurred when gemcitabine resistant PDOs were treated with VP plus gemcitabine (*n*:3; Figure S7). For this purpose, we evaluated cell viability for the combination at IC_90_ after 72h of treatment. For GBC-PDO1, we found an additive effect between VP and gemcitabine, which was reflected in a 50.1% reduction of cell viability (*p* = 0.041) compared with vehicle control. Nevertheless, the effects of this combination were not observed in GBC-PDO2 (Figure S7). The difference in the additive response may respond to the fact that GBC-PDO1 presents a higher nuclear intensity and percentage of positive cells for YAP1 than GBC-PDO2 (Figure 5C). These results suggest that YAP1 inhibition sensitizes cancer cells to gemcitabine and that gemcitabine plus VP could be an attractive candidate for combined therapy.

## 3. Discussion

The Hippo-YAP1 pathway has emerged as an important tumor suppressor kinase cascade with dysregulation leading to cellular transformation and the development of tumors [2]. The consequences of dysregulated Hippo pathway signaling on tumor initiation and progression have been reported in several tumors. However, only one study has explored the expression of YAP1 in GBC and the functional effects of *YAP1* knockdown by shRNA [6]. Here, we evaluated the status of key components of the Hippo pathway in GBC and the effects of targeted inhibition of YAP1 in this cancer. In an extended cohort of advanced GBC cases, we showed that expression of YAP1 and MST1 were reduced in GBC compared with chronic cholecystitis. Interestingly, we found that increased nuclear expression of YAP1 was associated with worse overall survival in subserosal (pT2) GBC. On the basis of these observations, we focused our research on the potential role of YAP1 in GBC.

YAP1 and TAZ are overexpressed in many primary tumors and increased nuclear staining of these proteins has been reported as a prognostic marker for poor survival in several types of cancer [6,20,21,22,23,24,25,26]. Here, we found that YAP1 and TAZ are highly expressed in over 60% of chronic cholecystitis (CC) and advanced tumors. We also found high expression levels of both proteins in non-lithiasic gallbladder epithelium tissue samples (5/5). Nuclear localization of YAP1 has been reported in normal mouse gallbladder organoids, which showed high expression of stem/progenitors cells markers (Lgr5, Prom1 (prominin-1/CD133), Sox4 and Msh2) and shared some of the regenerative traits observed in hepatoblast and precursor cells [27]. Confirming this claim, we observed a distinctive nuclear expression of YAP1 in a patchy pattern which was more markedly visible in the depth of the mucosal layer (Figure 1A and Figure S2). In cancer, nuclear expression of YAP1 and its role in carcinogenesis may be linked to the inflammatory state induced by interleukin-6, which directly induces the activation of activating Src family kinase [28]. The oncogenic role of nuclear YAP1 may also be linked to mechanical damage secondary to the accumulation of bile acids which activate YAP1 via a pathway dependent on the induction of the scaffold protein IQGAP1 [29]. On the other hand, in the liver, hyperactivated TAZ promotes inflammatory cytokine production and macrophage infiltration, playing a relevant role in tumor development [30]. Due to the relevance of inflammation in gallbladder cancer [31], we propose that nuclear translocation of YAP is a major event in the gallbladder carcinogenic process triggered by chronic inflammation. Based on our findings and the aforementioned studies, we propose that the expression of YAP1 in CC and in normal epithelia develops in a different biological context than during GBC. We believe that the expression of YAP1 in the context of CC occurs in response to an inflammatory and regenerative process triggered by chronic injury sustained by the presence of gallbladder stones. However, further evidence is warranted to confirm this claim.

MST1 is one of the main components of the cytoplasmic kinase module of the Hippo pathway. Together with MST2, these proteins phosphorylate and activate downstream kinases LATS1 and 2, SAV1 and MOB1. Activated LATS1/2 subsequently phosphorylate and inactivate the YAP1 oncogene and its paralogue TAZ [2,3]. Caspase-dependent cleavage or phosphorylation of its activation loop lead to MST1 translocation to the nucleus, which may be important for its pro-apoptotic functions [32]. MST1 activity can be affected by different molecular events, including a dysregulated crosstalk with oncogenic pathways and altered post-translational modifications at the protein level [10,33,34,35]. In our study, the immunohistochemical evaluation of MST1 expression confirmed a diminished expression in GBC compared with CC. The anticancer effects of MST1 have been reported for numerous types of cancer, including colorectal [34,35], breast [10,36,37] and lung cancers [38]. It should be noted that cytoplasmic expression of MST1 in cancer cells has been related with its function as a protective molecule acting in the canonical Hippo signaling pathway, through the inhibition of oncogenic YAP/TAZ [33]. In addition, MST1 can be cleaved by caspases which promotes its nuclear translocation, where it induces chromatin condensation and apoptosis [32]. In colorectal cancer, loss of cytoplasmic MST1 has been associated with higher T and/or N stage, higher tumor grade and poor prognosis [35]. Similarly, MST1 has been reported to be downregulated in breast cancer and proposed as a strong prognostic and predictive for disease-free survival [36,37]. Although we did not find a correlation between cytoplasmic expression and clinicopathological features, the nuclear expression of MST1 was significantly associated with pT stage and progression in our GBC cohort. Further studies are warranted to determine the tumor-suppressive properties of MST1 in GBC and its impact on patient survival.

The major function of the Hippo kinase cascade is to inhibit the oncogenic transcriptional module, which comprises the downstream transcriptional co-activator YAP1, TAZ and the family of transcription factors TEAD 1–4. YAP1 and TAZ proteins actively shuttle between the cytoplasm and the nucleus. Within the cytoplasm, they play a passive role in the regulation of specific signaling pathways. When localized in the nucleus, YAP1 and TAZ interact with DNA-binding transcription factors, particularly TEAD members, to regulate gene expression. Therefore, the functional activity of phosphorylated YAP1 and TAZ is inhibited through sequestration in the cytoplasm and/or proteasomal degradation [7]. Increasing evidence supports that YAP1 and TAZ are oncogenes in mammalian cells, empowering several of the key attributes of cancer cells such as proliferative advantage, cell invasion and migration, cancer stem cell traits, metastasis and drug resistance [39].

A previous study reported that nuclear expression of YAP1 was significantly increased in GBC and predicted poor survival in patients at advanced stages of the disease [6]. Our study showed that 63% of advanced GBC patients had high nuclear expression of YAP1. Moreover, high levels of nuclear YAP1 predicted poor prognosis for patients with a pT2 tumor who did not received additional therapy different from cholecystectomy. Currently, there are histopathologic prognostic markers for pT2 GBC patients, such as hepatic infiltration [40] and lymph node metastasis [41]. Both of these parameters have been associated with recurrence and worst survival following surgical resection of the tumor [40,41]. However, the assessment of immunohistochemical markers such as nuclear localization of YAP1 may prove to be a valuable addition for both patient risk stratification and therapeutic decision-making in this subgroup of patients. Withal, these findings are preliminary and warrant further validation in a prospective cohort.

To further evaluate the biological significance of the reduction of YAP1 and phospho-YAP expression levels, we inhibited YAP1 expression by siRNA knockdown and VP treatment, a newly identified YAP1 inhibitor [16] which anti-oncogenic effects have not previously been reported in GBC. Li et al., [6] reported that depletion of YAP1 using lentiviral delivery of shRNA significantly reduced cell proliferation, increased apoptosis and suppressed invasiveness of OCUG-1 and GBC-SD GBC cell lines, and also inhibited tumor growth in vivo. We observed similar results using YAP1 siRNA in ascites-metastases derived GBC cell lines GB-d1 and G-415, which showed decreased migration capacity. Furthermore, we provide evidence that pharmaceutical inhibition of YAP1 by VP is feasible for GBC advanced therapy. Our data showed that VP treatment reduced total YAP1 and phospho-YAP1 and inhibited the migration capacities of GBC cell lines. We further studied the inhibitory properties of VP in a pre-clinical model of ex-vivo patient-specific organoids derived from advanced GBC. PDOs showing high expression of nuclear YAP1 were resistant to gemcitabine but were sensitive to VP, while VP had an additive effect with gemcitabine in PDOs with high nuclear YAP1. These findings are relevant considering that PDOs can emulate clinical drug response [19,42]. However, it is necessary to validate these findings in an extended cohort of PDOs.

The complete mechanisms through which VP affects the oncogenic potential of tumor cells remain to be elucidated. It is known that VP inhibits the association between YAP1 and TEAD, presumably by binding to YAP1, changing its conformation and abrogating its interaction with TEAD [16]. Several studies have reported the antitumorigenic effects of VP. For instance, Brodowska et al. 2014 reported that VP treatment inhibits the growth, proliferation and viability of human retinoblastoma cell lines. The authors observed a concomitant downregulation of Axl, CTGF, survivin and OCT4 genes. The latter are reported to be controlled by the YAP-TEAD signaling pathway [43]. Complementary studies have shown that VP decreases YAP1 expression and affects its cellular location, promoting the translocation of YAP from the nucleus to the cytoplasm [9,44,45]. Other anti-tumor mechanisms described for VP are mediated by inhibiting autophagosome formation, the induction of apoptosis and elimination of stem-like cells [8,10]. In addition, considering the functional interplay between Hippo and Wnt/β-catenin, we believe that VP may also be affecting the expression of genes from this pathway. Consistently, it has previously been reported that VP may enhance the effects of gemcitabine and other anti-tumor drugs by inhibiting the expression of YAP1 [46,47,48], by inhibition of autophagy [8] or modulating lysosomal activity [49]. Hence, VP has been proposed as an effective chemosensibilizing agent. Nonetheless, the main goal of this manuscript is to highlight the potential therapeutic role of VP in GBC and not to explore its mechanisms. Therefore, other studies are needed in order to evaluate the molecular and biological mechanisms mediating the inhibitory effects of VP in gallbladder tumor cells.

Recently, a phase I/II clinical trial demonstrated that VP is a feasible and safe alternative for the photodynamic adjuvant treatment of inoperable pancreatic cancer patients [11]. Additionally, two phase III clinical trials evaluated the effectiveness and safety of Verteporfin in combination with light exposure for treatment of multiple basal cell carcinoma of the skin (NCT00049959). In accordance with our results, other recent reports have demonstrated the potential use of VP in vitro [10,50] and in vivo [8,12] as an effective targeted anticancer agent independent of light activation. However, more scientific evidence is necessary including in vivo animal studies to justify the potential use and effectiveness of VP in patients with advanced GBC.

## 4. Materials and Methods

### 4.1. Clinical Samples

This study was approved by the Ethics Committee of Faculty of Medicine (CEC-MedUC), Pontificia Universidad Católica de Chile (ID Nº 1600829038) and by the South-East Metropolitan Health Service Human Research Ethics Committee (Date of approval 16th May 2018). Tissue Microarrays (TMAs) containing 18 tissues of dysplasia, 28 of incipient cancers (13 mucosal and 15 muscular infiltration), 236 advanced gallbladder cancer derived from stage II to IV (pT2-T3-T4) and 173 chronic cholecystitis were used for immunohistochemical analysis. None of the included patients with advanced GBC received adjuvant chemotherapy or radiotherapy after surgical treatment [51]. Additionally, five non-lithiasic normal gallbladder epithelium from patients that underwent bariatric surgery and cholecystectomy were included. Fresh tissues for the establishment of GBC patient-derived organoids (PDO) were obtained from Hospital Clínico de la Pontificia Universidad Católica and Hospital Dr. Sótero del Río (Santiago, Metropolitan Region, Chile). In this case, all patients gave their informed consent. Samples were histologically analyzed to confirm percentage of cellularity and pathological diagnosis. For RNA extraction, fresh frozen tissues were preserved immediately in RNALater (Ambion Inc., Austin, TX, USA) and stored at −80 °C. Clinicopathologic features of patients with advanced GBC were obtained from medical records and are summarized in Table S1.

### 4.2. Immunohistochemistry Staining and Quantification

The immunohistochemical procedure was carried out as previously described [51]. Primary monoclonal rabbit anti-YAP (D8H1X, XP #14074, dilution 1:100) and polyclonal rabbit anti-MST1 (#3682, dilution 1:100) were purchased from Cell Signaling Technologies (Boston, MA, USA). Monoclonal anti-mouse TAZ (clone CL0371, #AMAB90730, dilution 1:100) was purchased from Sigma-Aldrich. Antibodies were diluted in Emerald antibody diluent (Cell Marque, Rocklin, CA, USA) and incubated for 30 min at room temperature. After three washes, 2 min each with PBS/0.01% Tween 20, the sections were incubated with secondary anti-rabbit antibody EnVision FLEX/HRP (Dako, Carpinteria, CA, USA) for 30 min. After three washes, 2 min each with PBS/0.01% Tween 20, the samples were developed with Vector^®^ NovaRED (Burlingame, CA, USA) or DAB+ Substrate chromogen system (Dako, Carpinteria, CA, USA), according to the manufacturer’s instructions. Slides were scanned using Aperio Digital Pathology Slide Scanner AT2 (Leica Biosystems, Wetzlar, Germany). The expression of YAP1 was scored as previously described [52]. The nuclear and cytoplasmic expression of MST1, TAZ and Survivin were scored by estimating proportion of tumor cells by multiplying the percentage of positive cells (P) by the intensity (I). Positive cells: 0, <10%; 1, 10–25%, 2, 25–50%; 3, 50–75%; 4, >75%. Staining Intensity: 1, Weak; 2, Moderate; 3, Strong. A final staining score of 0 was classified as negative, <4 low, ≥4 as high.

### 4.3. Cell Culture

The human GBC cell lines G-415, TGBC1TKB, TGBC2TKB were obtained from RIKEN BioResource Center (Ibaraki, Japan). NOZ was purchased from the health Science Research Resources Bank (Osaka, Japan) and GB-d1 was donated by Anirban Maitra (Department of Pathology, John Hopkins University School of Medicine, Baltimore, MD, USA). All cell lines were routinely tested for Mycoplasma by PCR and authenticated by the Dr. Justo Lorenzo Bermejo at the University of Heidelberg by short tandem repeat DNA profiling. They were used in the described experiments for a maximum of 10 passages after thawing.

### 4.4. YAP1 siRNA Transfection

For YAP1 siRNA studies, 2.5 × 10^5^ cells were transfected with a pool of four YAP1 siRNAs (FlexiTube GeneSolution GS10413) or the AllStars negative control (25 nM) from Qiagen (Valencia, CA, USA) using TransIT-siQUEST transfection reagent (3µL/6-well plaque) following the manufacturer’s instructions (Mirus Bio LLC, Madison, WI, USA).

### 4.5. Quantitative Real-Time PCR Analysis (qRT-PCR)

Total RNA was prepared using E.Z.N.A total RNA kit I (Omega Bio-tek, Norcross, GA, USA). To synthesize cDNA, purified total RNA (1 μg) was reverse-transcribed using the AffinityScript qRT-PCR cDNA Synthesis Kit (Stratagene, La Jolla, CA, USA). Quantitative expression analysis was performed using an oligonucleotide primer for the specific sequences of the transcripts (Table S5) and the Brilliant II SYBR Green qRT-PCR Master Mix (Stratagene, La Jolla, CA, USA). The *QARS* and *TFCP2* genes were used as internal controls. Thermal cycling and fluorescent detection were done using LightCycler480 (Roche, Diagnostics Corp, Indianapolis, IN, USA). Amplification data were analyzed using normalized ΔCq values and relative expression of mRNA was calculated as 2^(−ΔΔCq)^.

### 4.6. Verteporfin Treatments

For in vitro assays, cell lines were seeded on 10mm dishes. Once at 60-70% confluency, the cells were treated with Verteporfin (Sigma-Aldrich, #SML0534, St Louis, MO, USA) for 48 h. Verteporfin was dissolved in dimethyl sulfoxide (DMSO).

### 4.7. Western Blot Analysis

Protein samples were washed three times with cold 1× phosphate-buffered saline (PBS) and lysed in 1× RIPA buffer (Sigma-Aldrich, St Louis, MO, USA) supplemented with a protease inhibitor cocktail (Proteo-block, Fermentas, Glen Burnie, MD, USA). The nuclear cytoplasmic protein fractionation was performed with NE-PER Nuclear and cytoplasmic extraction reagents (Thermo Scientific, Rockford, IL, USA) according to the manufacturer´s protocol. Western blot protocol was performed as previously described [53]. The quantification of different specific bands was calculated by densitometry using ImageJ 1.6 version (National Institute of Health, Bethesda, MD, USA) and normalized to α-tubulin expression. Rabbit antibodies anti-YAP (D8H1X, XP #14074), anti-phospho-YAP Ser127 (D9W2I, #13008), anti-Histone H3 (D1H2, #4499), HRP-conjugated goat anti-rabbit (#7074) and anti-mouse (#7076) secondary antibodies were purchased from Cell Signaling Technologies Inc. (Danvers, MA, USA). Monoclonal mouse anti-α-tubulin was purchased from Invitrogen (B-5-1-2, #32-2500; ThermoFisher Scientific, MA, USA).

### 4.8. Transwell Cell Migration and Invasion Assays

The migration and invasion assays were carried out as previously described [53]. For migration assay, the cell lines (3 × 10^4^ cells per well) were exposed to 500 nM or 1250 nM of Verteporfin and 0.004% DMSO (as control) for 24 h at 37 °C using 10% fetal bovine serum in the lower chamber as a chemoattractant. Invasion assays were performed with 5 × 10^4^ cells as for the migration assays, except inserts were pre-coated with Matrigel (#356230; Corning, NY, USA) at a concentration of 20 μg and the incubation period was extended to 48 h.

### 4.9. Gallbladder Cancer Patient-Derived Organoids Culture (GBC-PDOs)

GBC-PDOs cultures were established from tumor tissue samples of patients diagnosed with moderated differentiated and pT3 N0/N1 Mx stage (Eight edition of American Joint Committee on Cancer (AJCC)) advanced tubular GBC. Gallbladder tumor organoids were generated as previously described [54]. For organoids cultures, dissociated cells were washed in 1× DPBS and embedded in growth factor reduced Matrigel (#356231, Corning, NY, USA) and cultured in complete media (Intesticult (#6005, Stemcell Technologies, Vancouver, BC, Canada), 0.5 μmol/L A83-01 (#SML0788; Sigma-Aldrich), 100 ng/mL, FGF10 (#100-26, PeproTech, Inc., Rocky Hill, NJ, USA), 100 ng/mL HGF (#100-39; Peprotech), 10 nmol/L Gastrin I (#G9145; Sigma), 10 mmol/L N-acetyl-l-cysteine (#A9165; Sigma-Aldrich) 10 mmol/L nicotinamide (#N0636; Sigma-Aldrich), 1× B27 supplement (#17504-044; Gibco, Thermo Fisher Scientific, Waltham, MA, USA), 1× N2 supplement (#175020-048; Gibco), 1 mg/mL Primocin (#ant-pm-1; InvivoGen, San Diego, CA, USA) and 10.5 μmol/L Y-27632 (#Y0503, Sigma-Aldrich). Organoids were passaged via mechanical dissociation and passage was performed weekly with a 1:3 ratio.

### 4.10. Drug Assay in Patient-Derived Organoids

Organoids were mechanically dissociated before being resuspended in 2% matrigel/growth media seeded (500 cells/well) and dispensed in 96-well plates (Costar #3610; Corning, NY, USA). Cells were incubated at 37 °C/5% CO_2_ overnight and then exposed to gemcitabine (Calbiochem #504594, Merck-Millipore, Darmstadt, Germany) using a 11-point dose-response curve with a 1:3 dilution series from 10 μM to 0.2 nM. After 72 h, relative viability was measure by CellTiter-Glo 3D cell viability assay (#G9683; Promega, MA, USA). Dose-response curves were performed in technical and biological (different passage) triplicates. Drug concentrations (molar units) were converted to log-scale and IC_50_ and IC_90_ concentrations were calculated by using GraphPad Prism (San Diego, CA, USA).

### 4.11. Statistical Analysis

Wilcoxon–Mann–Whitney U-test was used to assess the qRT-PCR and immunohistochemical staining score. The associations between protein expression and clinicopathological variables were fist examined with chi-squared tests. Jonckheere-Terpstra test was performed to evaluate the statistical significance of the observed trend in immunohistochemistry (IHC) scores in GBC progression through CC, dysplasia, incipient and advanced GBC samples. For advanced GBC, Kaplan–Meier survival curves were plotted for patients grouped according to their protein expression levels. Overall survival (OS) was defined as the time from the date of the surgery for GBC to death, whether cancer-related or not. Log-rank (Mantel–Cox) test was used to determine differences between survival curves. Statistical analysis of in vitro was performed by one-sample t-test. Data were analyzed using GraphPad Prism 5 software for Windows (GraphPad, San Diego, CA, USA), SPSS version 17.0 (SPSS, Chicago, IL, USA) and R statistical programming environment as implemented in Rstudio Version 1.0 (Inc., Boston, MA, USA). For all tests, a *p*-value < 0.05 was considered statistically significant.

## 5. Conclusions

In summary, we identified that key components of the Hippo-YAP1 signaling pathway are dysregulated in GBC and that high nuclear levels of YAP1 predict worse prognosis in patients with pT2 gallbladder cancer. Moreover, Verteporfin, an inhibitor of the Hippo-YAP1 pathway, showed efficient cytotoxicity against gemcitabine-resistant gallbladder patient-derived organoids (PDOs). Taken together, our results suggest that the Hippo-YAP1 pathway may be a candidate for targeted therapy in advanced GBC patients that not respond to gemcitabine treatment. However, further validation involving a larger sample set is required before possible implementation into clinical practice.

## Supplementary Materials

The following are available online at https://www.mdpi.com/2072-6694/12/4/778/s1, Table S1: Clinicopathologic characteristics and protein expression of MST1, YAP1 and TAZ in tumor and non-neoplastic gallbladder tissues, Figure S1: Expression of key Hippo-YAP1 signaling pathway-related genes in GBC patients, Figure S2: Expression of YAP1 in chronic cholecystitis patients- derived organoids by immunohistochemical staining, Table S2: Analysis of clinical, pathological and immunohistochemical markers by a multiple correlations test in a logarithmic multinomial model, Table S3: Analysis of immunohistochemical markers by a multiple correlations test in a logarithmic multinomial model, Table S4: Variables used in the equation of Cox proportional hazard regression analysis, Figure S3: Standardization of YAP1 siRNA conditions in gallbladder cancer cells, Figure S4: Invasion analysis in inserts of cell lines post-treatment with siRNA non-target control and siRNA YAP1, Figure S5: Verteporfin treatment did not affect cell viability at 24, 48 and 72 h in GB-1 and G-415 cancer cell lines, Figure S6: Verteporfin treatment did not affect apoptosis or cell cycle in GB-1 and G-415 cancer cell lines, Figure S7: Cytotoxicity of gemcitabine and/or cisplatin plus VP in resistant-GBC-PDOs 1 and 2, Figure S8: Original blots of the Western blot shown in Figure 3 and Figure 4, Table S5: RT-qPCR primers list of human YAP1 and control genes.
