# Supplementary material for: Hippo-YAP1 Is a Prognosis Marker and Potentially Targetable Pathway in Advanced Gallbladder Cancer
Source: Cancers (Basel). 2020 Mar 25;12(4):778. doi: 10.3390/cancers12040778 (PMC7226626; doi:10.3390/cancers12040778)
Supplement: Supplementary file 1 [file cancers-12-00778-s001.zip › cancers-736842-Supplementary materials.docx]

Supplementary Materials: Hippo-YAP1 Is a Prognosis Marker and Potentially Targetable Pathway in Advanced Gallbladder Cancer

Patricia García, Lorena Rosa, Sergio Vargas, Helga Weber, Jaime A. Espinoza, Felipe Suárez, Isabel Romero-Calvo, Nicole Elgueta, Vanessa Rivera, Bruno Nervi, Javiera Obreque, Pamela Leal, Eduardo Viñuela, Gloria Aguayo, Sabrina Muñiz, Alfredo Sagredo, Juan C. Roa and Carolina Bizama

**Figure S1.** Expression of key Hippo-YAP1 signaling pathway-related genes in GBC patients. Relative mRNA expression levels in chronic cholecystitis (CC), advanced gallbladder cancer cases (T) and gallbladder cancer cell lines (CL). *QARS* and *TFCP2* were used as endogenous controls. The data are expressed as scatter plot and the comparison between CC and T expression data was performed by Wilcoxon-Mann-Whitney U-Test. *p*-value: * < 0.05, ** < 0.01, ns, non-significant.


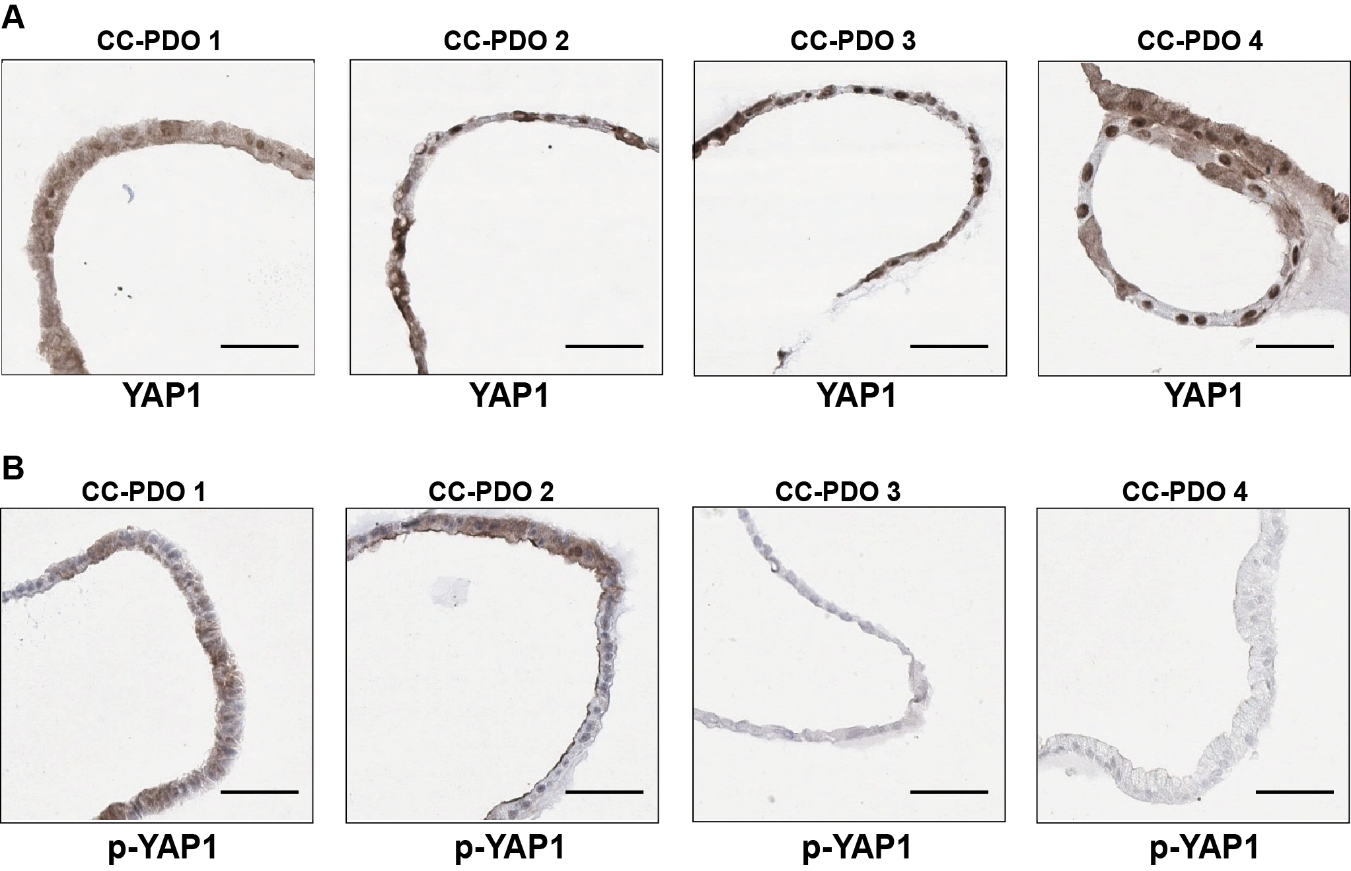


**Figure S2.** Expression of YAP1 in chronic cholecystitis patients- derived organoids by immunohistochemical staining. Scale bar: 50 µm.

**Table S2.** Analysis of clinical, pathological and immunohistochemical markers by a multiple correlations test in a logarithmic multinomial model.

| **Source** | ***Β*** | ***SE B*** | **Wald χ^2^** | ***p*** | ***OR*** | **95% CI OR** |
| --- | --- | --- | --- | --- | --- | --- |
| Intersection | −13.511 | 4.621 | 8.550 | 0.003 |  |  |
| Age | −0.008 | 0.022 | 0.146 | 0.702 | 0.992 | [0.951, 1.035] |
| Sex | 0.504 | 0.723 | 0.485 | 0.486 | 1.655 | [0.401, 6.824] |
| Ethnicity | −0.027 | 0.602 | 0.002 | 0.964 | 0.973 | [0.299, 3.167] |
| Infiltration | 3.941 | 1.257 | 9.837 | 0.002 | 51.485 | [4.386, 604.408] |
| Differentiation | 0.005 | 0.443 | 0.000 | 0.990 | 1.005 | [0.422, 2.398] |
| Nuclear YAP1 | 0.552 | 0.370 | 2.224 | 0.136 | 1.736 | [0.841, 3.586] |
| Cytoplasmic YAP1 | 0.008 | 0.103 | 0.006 | 0.936 | 1.008 | [0.824, 1.234] |
| Nuclear Survivin 1 | 0.055 | 0.084 | 0.428 | 0.513 | 1.056 | [0.897, 1.244] |
| Cytoplasmic Survivin 1 | −0.047 | 0.115 | 0.169 | 0.681 | 0.954 | [0.761, 1.195] |
| Nuclear GPCR5A | 0.143 | 0.120 | 1.420 | 0.233 | 1.153 | [0.912, 1.458] |
| Cytoplamic GPCR5A | 0.201 | 0.147 | 1.868 | 0.172 | 1.223 | [0.916, 1.631] |
| Nuclear MST1 | −0.158 | 0.154 | 1.048 | 0.306 | 0.854 | [0.632, 1.155] |
| Cytoplasmic MST1 | −0.060 | 0.172 | 0.120 | 0.729 | 0.942 | [0.672, 1.320] |
| Nuclear P53 | 0.081 | 0.057 | 2.005 | 0.157 | 1.084 | [0.969, 1.213] |
| Nuclear Survivin 2 | −0.035 | 0.113 | 0.095 | 0.757 | 0.966 | [0.773, 1.206] |
| Cytoplasmic Survivin 2 | −0.777 | 0.558 | 1.935 | 0.164 | 0.460 | [0.154, 1.374] |
| Nuclear TAZ | −0.052 | 0.108 | 0.231 | 0.631 | 0.949 | [0.768, 1.174] |
| Cytoplasmic TAZ | 0.019 | 0.104 | 0.035 | 0.852 | 1.020 | [0.832, 1.249] |

B: unstandardized regression weight; SE B: multiple linear regression; Wald χ^2^: statistical test for the individual predictor variable; *p*: *P*-value used to determine statistical significance; OR: odds ratio and CI: confidence interval.

**Table S3.** Analysis of immunohistochemical markers by a multiple correlations test in a logarithmic multinomial model.

| **Source** | ***Β*** | ***SE B*** | **Wald χ^2^** | ***p*** | ***OR*** |
| --- | --- | --- | --- | --- | --- |
| Nuclear GPCR5A | −0.092 | 0.317 | 0.084 | 0.772 | 0.912 |
| Cytoplamic GPCR5A | 0.418 | 0.311 | 1.808 | 0.179 | 1.519 |
| Nuclear MST1 | 0.365 | 0.366 | 0.998 | 0.318 | 1.441 |
| Cytoplasmic MST1 | −0.468 | 0.350 | 1.784 | 0.182 | 0.626 |
| Nuclear P53 | 0.174 | 0.323 | 0.292 | 0.589 | 1.191 |
| Nuclear Survivin 2 | 0.538 | 0.445 | 1.466 | 0.226 | 1.713 |
| Cytoplasmic Survivin 2 | 0.384 | 0.333 | 1.326 | 0.250 | 1.468 |
| Nuclear TAZ | −0.408 | 0.430 | 0.899 | 0.343 | 0.665 |
| Cytoplasmic TAZ | 0.233 | 0.401 | 0.338 | 0.561 | 1.263 |
| Nuclear YAP1 | −0.639 | 0.337 | 3.597 | 0.048 | 0.528 |
| Cytoplasmic YAP1 | −0.243 | 0.329 | 0.546 | 0.460 | 0.784 |

B: unstandardized regression weight; SE B: multiple linear regression; Wald χ^2^: statistical test for the individual predictor variable; *p*: *P*-value used to determine statistical significance and OR: odds ratio.

**Table S4.** Variables used in the equation of Cox proportional hazard regression analysis.

| **Variables in the equation** | ***Β*** | ***SE B*** | **Wald χ^2^** | ***p*** | ***OR*** |
| --- | --- | --- | --- | --- | --- |
|  | −0.195 | 0.226 | 0.748 | 0.387 | 0.823 |

B: unstandardized regression weight; SE B: multiple linear regression; Wald χ^2^: statistical test for the individual predictor variable; *p*: *P*-value used to determine statistical significance and OR: odds ratio.


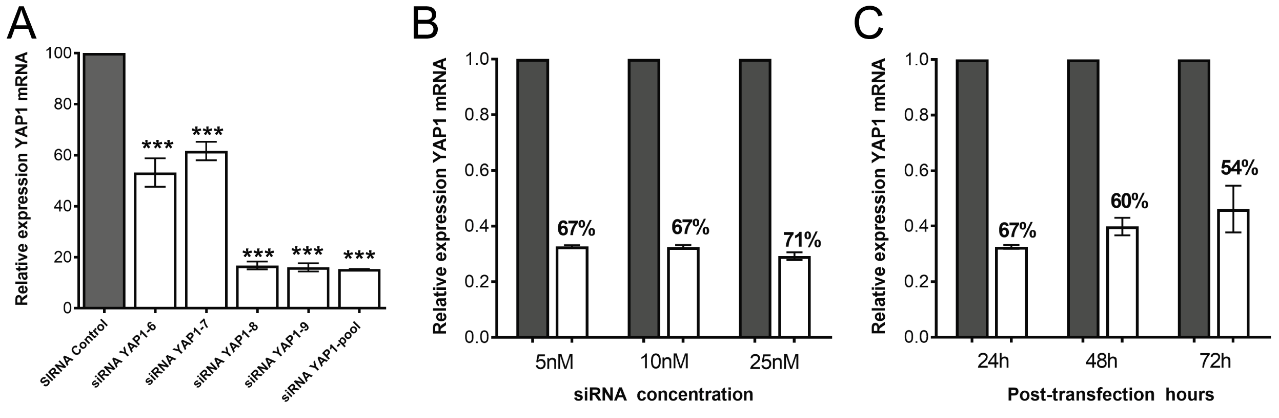


**Figure S3.** Standardization of YAP1 siRNA conditions in gallbladder cancer cells. (**A**), Relative expression of YAP1 (% of siRNA control) for 4 individual siRNAs separately and siRNA pool. One-way ANOVA with Dunnett comparison test (*** *p* < 0.001). YAP1 knockdown percentage by siRNA pool: (**B**), at different siRNA pool concentrations and (**C**)**,** different post-transfection time points.


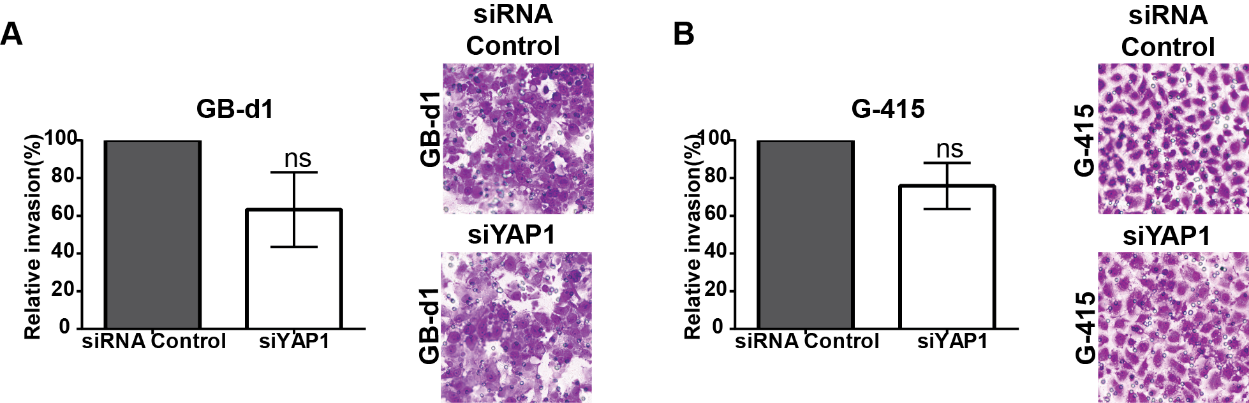


**Figure S4.** Invasion analysis in inserts of cell lines post-treatment with siRNA non-target control and siRNA YAP1. (A) GB-d1 and (B) G-415 cell line at 48 h. The experiment was repeated in triplicate for each cell line. The data are expressed as the mean ± SD. ns. = not significant by one-sample *t*-test. Magnification: 20×.


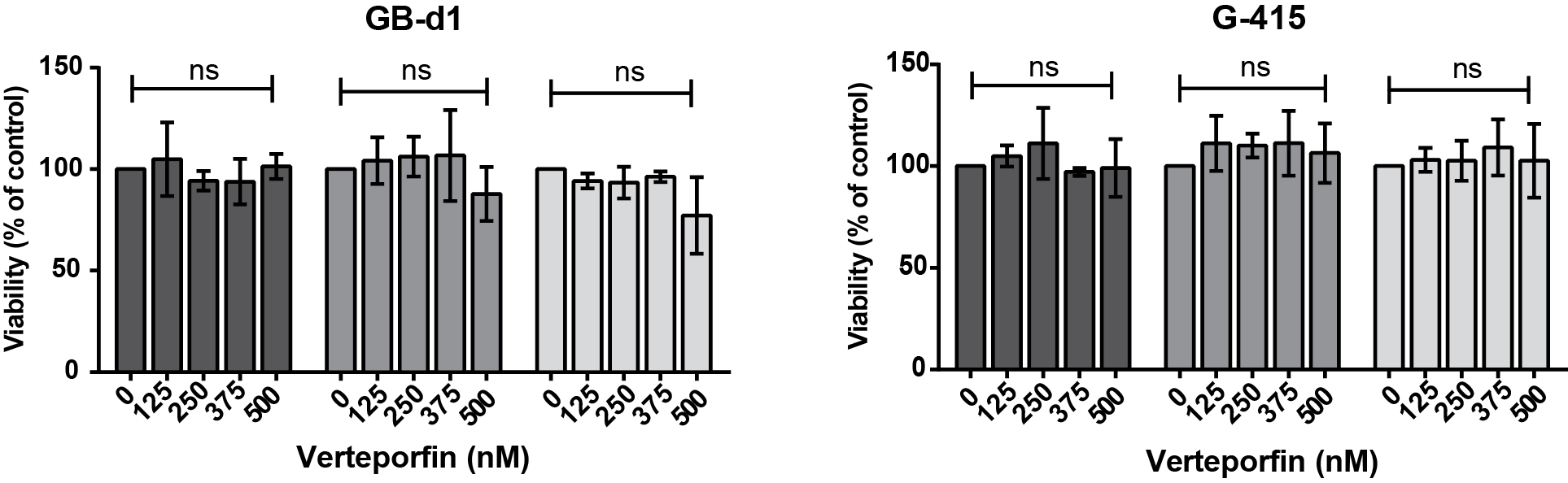


**Figure S5.** Verteporfin treatment did not affect cell viability at 24, 48 and 72 h in GB-1 and G-415 cancer cell lines. Viability of GB-d1 and G-415 cells (3500 each well) by MTS analysis after treatment with DMSO as a control (0) or different doses of VP (125, 250, 375 and 500 nM). The experiment was repeated in triplicate for each cell line. Error bars indicated mean ± SD. n.s.: no significant by 2-way ANOVA with Bonferroni´s multiple comparison test.


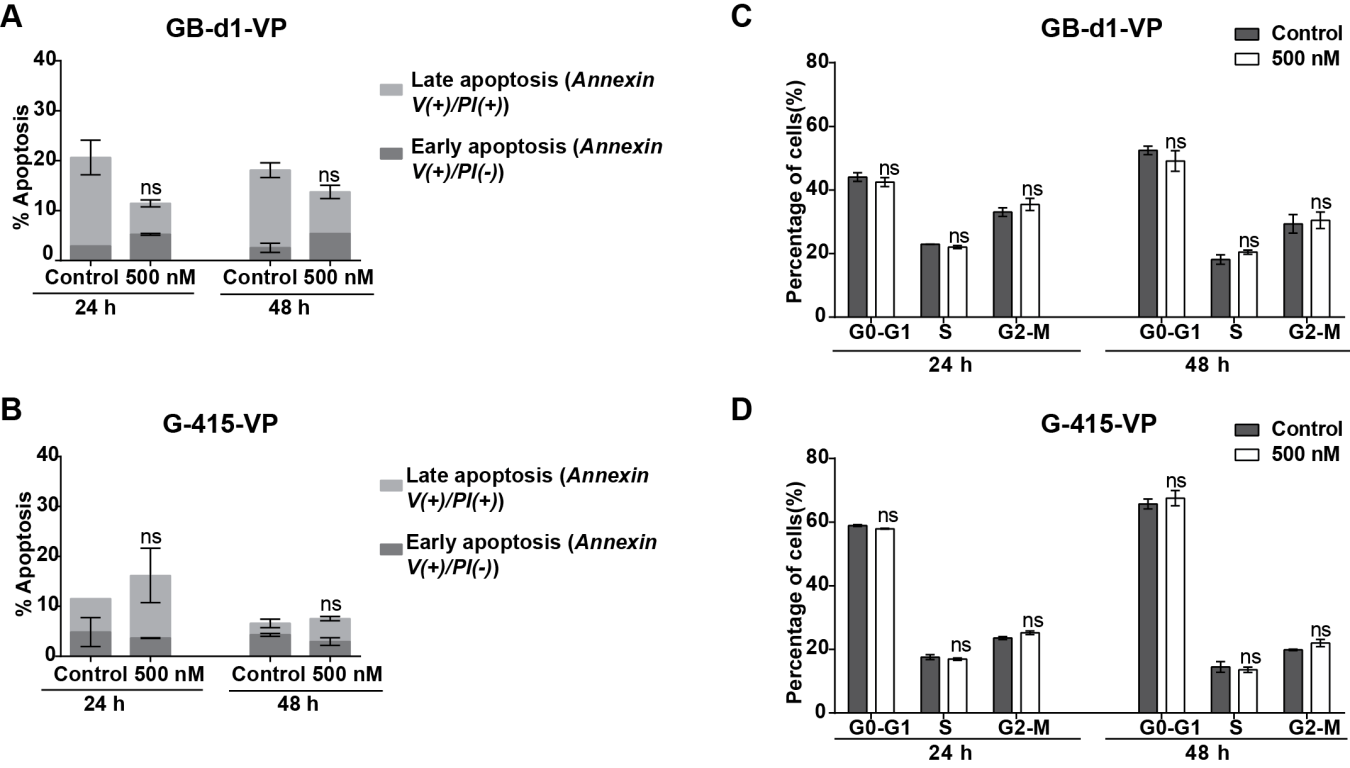


**Figure S6.** Verteporfin treatment did not affect apoptosis or cell cycle in GB-1 and G-415 cancer cell lines. Percentage of apoptosis of: (**A**), GB-d1 and (**B**), G-415 at 24- and 48-hours post-treatment., Percentage of cells at each phase of cell cycle in (**C**), GB-d1 and (**D**), G-415. Results of apoptosis and cell cycle analyses are represented as Mean ± SD from 2 independent experiments. Results were analyzed by unpaired *t*-test with n.s. = not significant.


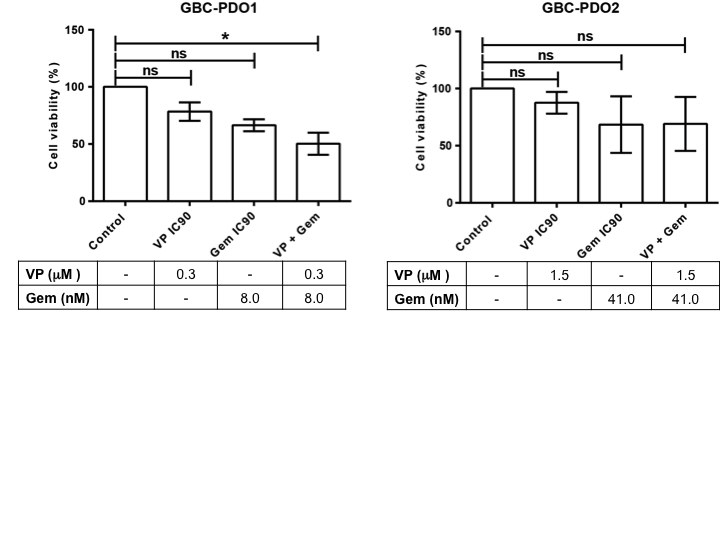


**Figure S7.** Cytotoxicity of gemcitabine and/or cisplatin plus VP in resistant-GBC-PDOs 1 and 2. Results are represented as Mean ± SD from 3 independent experiments. *p* < 0.05 by by Kruskal Wallis with Dunn's post hoc test. N.s. = not significant


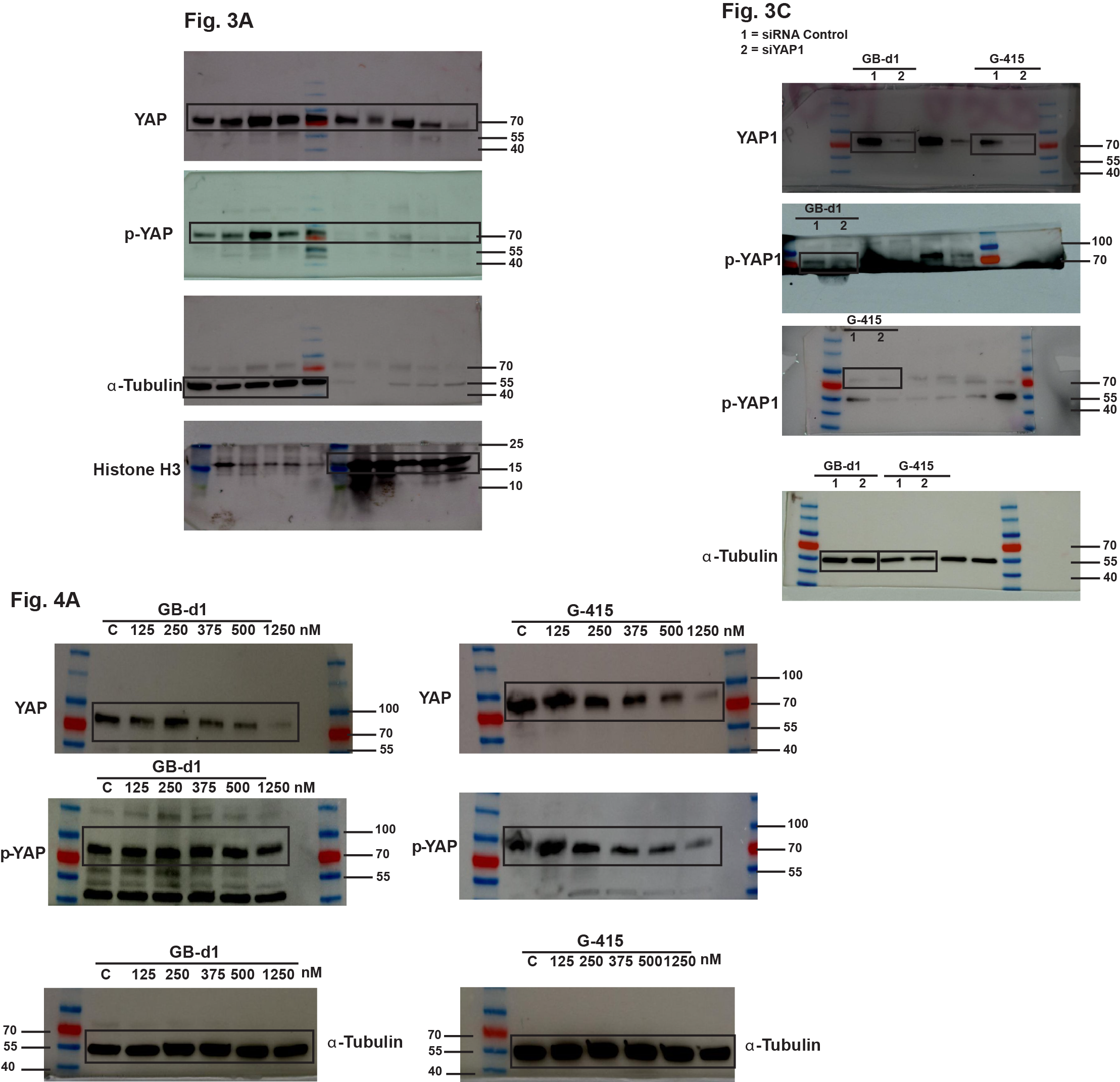


**Figure S8.** Original blots of the Western blot shown Figure 3 and 4.

**Table S5.** RT-qPCR primers list of human Hippo-YAP1 signaling pathway and control genes^a^.

| **Symbol** | **Primer 5´-3´** | **pb** | **Tm** | **Amplicon** | **Gen_ ID** |
| --- | --- | --- | --- | --- | --- |
| **YAP1_Forward** | GCCACTGCAGATGGAGTTTTAG | 22 | 56.0 | 133pb | 10413 |
| **YAP1_Reverse** | GGCACTCCTTCCAAGTAGCTTT | 22 | 56.9 |  |  |
| **TAZ_Forward** | CCCGCTTTGGACAGAAAATCAC | 22 | 56,8 | 126pb | 25937 |
| **TAZ_Reverse** | GAATGAAGTCCGTCAGGGCTTT | 22 | 57,2 |  |  |
| **STK3_Forward** | GCATTGGGACCAGTCATTTCCT | 22 | 57.2 | 123pb | 6788 |
| **STK3_Reverse** | TATGCTGGGTGGCACAGTGAA | 21 | 58.4 |  |  |
| **STK4_Forward** | TGCATGGATGAGTGGATGAGTG | 22 | 56.8 | 104pb | 6789 |
| **STK4_Reverse** | ACAGTCTCAAGGAGTCAAGGCT | 22 | 57.3 |  |  |
| **SAV1_Forward** | ACCAGCCACAGCAAACTGAA | 20 | 57.0 | 101pb | 60485 |
| **SAV1_Reverse** | GGCTCGTGCGTAAACCTGAA | 20 | 57.7 |  |  |
| **LATS1_Forward** | ACTGTGATGCCACCTGTTGCT | 21 | 59.0 | 138pb | 9113 |
| **LATS1_Reverse** | GCTTGGCTGATCCTCTTTGCT | 21 | 57,4 |  |  |
| **LATS2_Forward** | TGCTCAATGTCACGGAGACCA | 21 | 58,2 | 187pb | 26524 |
| **LATS2_Reverse** | ACACACCAGACGTCGGAAATC | 21 | 57,2 |  |  |
| **MOB1A_Forward** | GCGTCTGTTCAGGGTTTATGCC | 22 | 58,5 | 148pb | 55233 |
| **MOB1A_Reverse** | TGCCAGCTCACGCCTATCAATC | 22 | 59,4 |  |  |
| **MOB1B_Forward** | TGCACATGGTGTTACATGGCTA | 22 | 56,6 | 146pb | 92597 |
| **MOB1B_Reverse** | GCTGCAGAACAAAGCACTGA | 20 | 56 |  |  |
| **TFCP2_Forward** | CCCTTGCCAGATCAGCCAGATTT | 23 | 59.3 | 168pb | 5859 |
| **TFCP2_Reverse** | ACGCCGCACTCCTACTTCAGTAT | 23 | 59.8 |  |  |
| **QARS_Forward** | ACCTGAACCTGGCATCACTACA | 22 | 57.6 | 100pb | 7024 |
| **QARS_Reverse** | CCAAGACGCTCAAACTGGAACT | 22 | 57.5 |  |  |

^a^: Amplifications conditions were as follows: one cycle 95 °C for 10 min, followed by 40 cycles of amplification at 95 °C for 15 s, 60 °C for 15 s and 72 °C for 15 s.

| 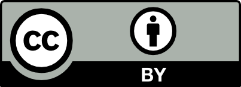 | © 2020 by the authors. Licensee MDPI, Basel, Switzerland. This article is an open access article distributed under the terms and conditions of the Creative Commons Attribution (CC BY) license (http://creativecommons.org/licenses/by/4.0/). |
| --- | --- |
